# Effects of cognitive training on cognitive function in patients after cardiac surgery: A systematic review and meta-analysis of randomized controlled trials

**DOI:** 10.1097/MD.0000000000040324

**Published:** 2024-11-01

**Authors:** Rongxiang Zhang, Chenyang Zhu, Shiqi Chen, Feng Tian, Pingping Huang, Yuan Chen

**Affiliations:** a Xiamen Cardiovascular Hospital, Xiamen University, Xiamen, China; b School of Nursing, Fujian University of Traditional Chinese Medicine, Fuzhou, China.

**Keywords:** cardiac surgery, cognitive training, meta-analysis, postoperative cognitive dysfunction, systematic review

## Abstract

**Background:**

Postoperative cognitive deficits frequently occur in patients undergoing cardiac surgery, leaving them with reduced cognitive function. Cognitive training has been shown to improve cognitive function, however, the role in patients after cardiac surgery is unclear. In this study, we aimed to evaluate the effectiveness and safety of cognitive training in patients undergoing cardiac surgery.

**Method:**

A systematic search of PubMed, Embase, Cochrane Library, CINAHL, Ovid Medline, Web of Science, CNKI, and Wanfang was conducted until March 2024. The risk of bias was assessed using the Cochrane Risk of Bias Tool. Data were meta-analyzed using RevMan 5.4 software. Potential bias and reliability of evidence were fairly assessed by using the Cochrane risk of bias method and the GRADE evidence grading method.

**Results:**

A total of 16 studies involving 1335 cardiac surgery patients were included in this study. Compared with the control group, the cognitive training group had a significantly lower incidence of postoperative cognitive dysfunction (RR 0.35, 95% CI 0.18–0.65, *P* = .001), significantly improved cognitive function (MD 2.54, 95% CI 1.27–3.81, *P* < .001), and a significantly higher quality of life-mental component (MD 5.22, 95% CI 2.32–8.13, *P* < .001), anxiety (MD −6.05, 95% CI −10.96 to −1.15, *P* = .02) and depression (MD −3.97, 95% CI −7.15 to −0.80, *P* = .01) were significantly improved between groups. However, the differences were not statistically significant for postoperative delirium (RR 1, 95% CI 0.38–2.65, *P* = 1.00) and postoperative hospitalization (MD −0.95, 95% CI −2.90 to 1.00, *P* = .34).

**Conclusions:**

The present study, based on a low to moderate quality of evidence, suggests that cognitive training improves cognitive functioning, reduces the incidence of postoperative cognitive dysfunction, and has a positive impact on anxiety and depression in patients undergoing cardiac surgery. However, current evidence does not allow for the determination of effects on quality of life, postoperative delirium, and postoperative length of stay.

## 1. Introduction

Cardiovascular disease has long been the leading cause of death globally, and in 2020 has already caused 19.05 million deaths worldwide.^[1]^ Advancements in technology and innovation of novel instruments currently define the evolving landscape of cardiac operations, translating into enhanced safety margins amidst the perioperative phases and steadily declining fatality tolls in the immediate aftermath of cardiac surgeries. As a consequence, practitioners are empowered to render premium quality operative services for cardiovascular sufferers while simultaneously mitigating procedure-associated jeopardy.^[2,3]^ Although accomplishments characterizing cardiac surgery continue to climb, postoperative hindrances still surface, exacting heavy consequences upon recuperative trajectories and operational payoffs.^[4,5]^ Among these hurdles lies postoperative cognitive dysfunction (POCD), constituting a widespread challenge faced by recipients navigating cardiac surgical recoveries.^[6]^ Takafumi Oyoshi et al^[7]^ examined a subset of seventy-one individuals, aged between forty-six and sixty-four years, undergoing voluntary cardiac surgeries, divulging that above a third of middle-aged surgical patients later suffered from POCD incidence. Congruous discoveries appeared in the Kristiina Relander et al^[8]^ steered a study, whereupon one hundred antecedent Coronary Artery Bypass Graft operation aspirants demonstrated deteriorated cognitive prowess amounting to forty-seven percentage points within the tri-monthly follow-up window.

POCD signifies a temporal juncture following surgical procedures wherein patients register substantial losses in cognitive capability, spanning areas such as concentration, mnemonics, and processing velocity, alongside descent aptitudes.^[9]^ Instances of POCD carry implications for patient rehabilitation after cardiac operations, drawing out hospitalizations, inflating healthcare expenses, generating economic strain,^[10]^ and influencing emotive responses. Moreover, persistent cognitive incapacitation raises concerns about heightened vulnerabilities to lasting confusion, premature mortality,^[11,12]^ and dwindling lifestyle enjoyment.^[8]^

Cognitive Training represents a non-pharmaceutical strategy predicated on specialized, behaviorally constrained cognitive or socio-emotional pedagogical experiences, presented iteratively and adaptively to promote neural network efficiency and augment broader cognitive capabilities for patients.^[13]^ Empirical evidence supports the efficacy of cognitive training in improving cognitive function across diverse demographics, including but not limited to, patients suffering neurodegenerative decline, aging cohorts, and those diagnosed with Parkinson’s Disease.^[14–16]^ With increasing regularity, randomized controlled trials have turned their gaze towards studying the influence of cognitive training on patients post-cardiac surgery, though arriving at conflicting conclusions. Notwithstanding this surge in investigatory efforts, no formalized systematic overview has attempted to summarize and critique the impact of cognitive training on patients undergoing cardiac surgery nor explore how compliance may mediate the effectiveness of fall-prevention interventions. Guided by this gap in current knowledge, our multidisciplinary team resolved to undertake a comprehensive literature survey, complemented by a meticulous quantitative synthesis aimed at critically evaluating the emerging body of evidence.

## 2. Methods

This systematic review was conducted according to the Preferred Reporting Items for Systematic Reviews and Meta-analyses (PRISMA). The protocol of this systematic review was registered at PROSPERO (ID: CRD42024526548).

### 2.1. Search strategy

The present study implemented a rigorous literature search, accessing digital libraries such as PubMed, Embase, Cochrane Central Register of Controlled Trials (CENTRAL), Cumulative Index to Nursing and Allied Health Literature (CINAHL), Ovid Medline, Web of Science, China National Knowledge Infrastructure (CNKI), and Wanfang databases. The search covered the entirety of each repository’s historical record up until March 2024. The PubMed search strategy is included in the online supplemental material (Appendix S1, Supplemental Digital Content, http://links.lww.com/MD/N859).

### 2.2. Inclusion criteria

Article selection transpired via stringent, mutually agreed-upon inclusion and exclusion criteria, implemented separately by two researchers, with disagreements addressed via consultation or resorting to arbitration by a third researcher whenever required. Duplicate entries were eradicated initially through the employment of Note Express software, succeeded by an inaugural screening stage discarding subject irrelevancies solely based on title inspection. Abstracts belonging to the residual collection then underwent rigorous vetting, permitting entry based on prespecified requirements, before conducting a terminal full-text evaluation of fitting prospects.

#### 2.2.1. Study type

The type of study experiment was a randomized controlled trial. The literature was published in English and Chinese languages.

#### 2.2.2. Types of participants

Eligible participants consisted of individuals satisfying the following mandatory conditions: attainment of majority status equivalent to eighteen years of age or higher; and verification of previous history or scheduled plans concerning invasive cardiac procedures.

#### 2.2.3. Intervention type

Included within the study interventions lay cognitive training, formally characterized as repeated drills requiring active engagement in intellectually demanding assignments, supplemented by strategic guidance or guided practice sessions leveraging computerized platforms or traditional pen-and-paper approaches.

#### 2.2.4. Comparison type

Routine care with no cognitive training included.

#### 2.2.5. Outcome

Selected studies were mandated to report on at least one of the ensuing outcomes: POCD, cognitive function, depression, anxiety, quality of life, postoperative delirium, and postoperative hospitalization time.

### 2.3. Exclusion criteria

This study opted to exclude research falling under the following categories: denial of access to full-length documents; cases of redundant publications; depictions marred by data gaps or deficiencies; and low methodological quality evaluations evident in the literature (all 7 items in Cochrane’s risk of bias instrument are high risk).

### 2.4. Data extraction and outcomes

In alignment with PRISMA protocols, two trained researchers independently harvested pertinent data via standardized extraction templates, itemizing study features like author names, headlines, regional provenance, and circulation dates. Other highlights included population characteristics, particularly age distribution, sex ratio, and sample size. Intervention specifics, including aspects such as type, duration, and frequency. Primary outcome indicators were extracted including POCD, cognitive function, and postoperative delirium. Secondary outcome indicators included quality of life, depression, anxiety, and length of postoperative hospitalization. If individual studies included were inconsistent in their data presentation, this study was transformed using statistical methods if necessary.

### 2.5. Assessment of study quality

Quality appraisal of synthesized investigations unfolded by the strictures outlined within the Cochrane Handbook for Systematic Reviews of Interventions, as published by the Cochrane Collaboration. Seven major spheres merited careful review, commencing with production of random sequence generation; implementation of allocation concealment; blinding of investigators and subjects; objectivity of outcome assessments; integrity of data regarding primary outcomes (attrition rate); potential for selective reporting of findings; and existence of any additional biases. Two researchers operated independently to gauge the methodological excellence of integrated studies. When consensus faltered, recourse was sought through adjudication from a seasoned third party.

### 2.6. Statistical analysis

Meta-analytic calculations were executed with the aid of the RevMan 5.4 platform. Mean difference (MD) was used as the effect indicator for the measurement data, and relative risk (RR) was used as the effect indicator for the count data. Investigation of heterogeneity transpired through the *I*^2^ statistic; if *I*^2^ values fell below 50%, a fixed-effects model was deemed sufficient, otherwise, a random-effects model supplanted it. Robustness checks took the shape of sensitivity analyses, maintaining a testing threshold of α = 0.05; *P* values less than .05 triggered declarations of statistical significance. Research that couldn’t lend itself to combination warranted descriptive syntheses.

To establish the soundness of evidence buttressing this investigation, each outcome parameter underwent meticulous evaluation rooted in the principles espoused by the Grading of Recommendations Assessment, Development, and Evaluation (GRADE) framework.

## 3. Results

### 3.1. Study selection

**Figure 1.**
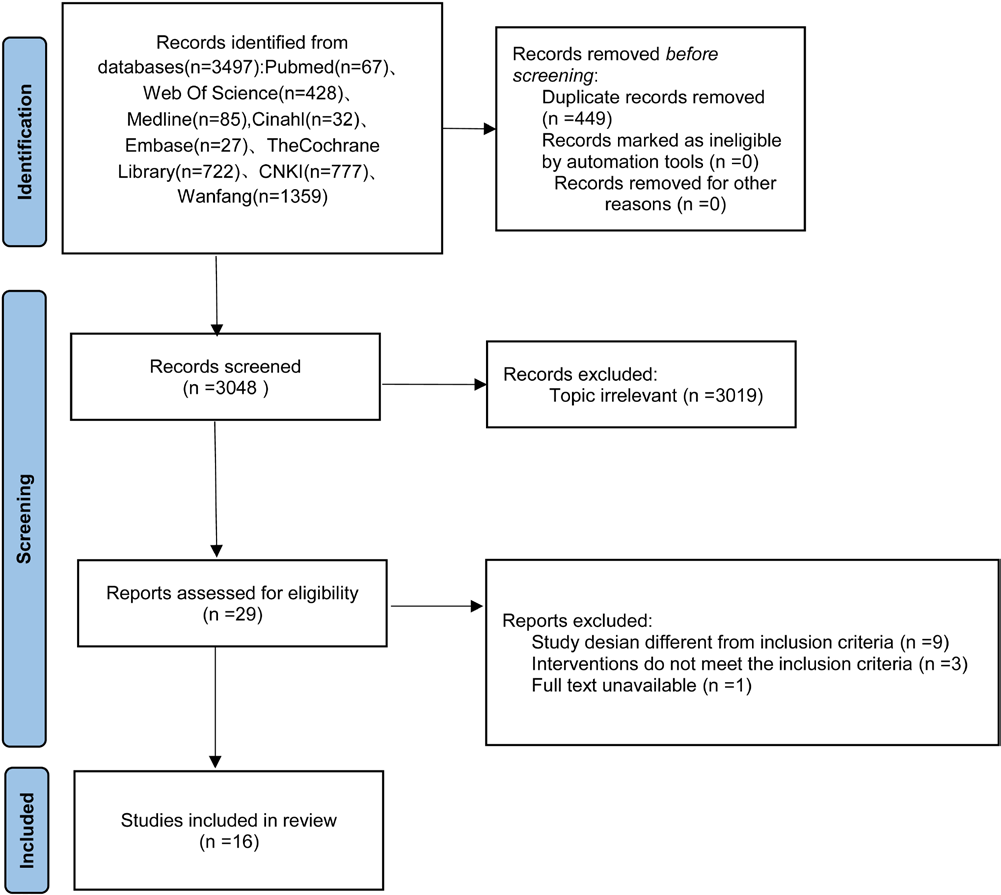
Flowchart of the literature screening process.

Strict adherence to an established search strategy and screening protocol enabled the successful acquisition and filtering of pertinent literature. Depicted in Figure 1 is the literature retrieval and screening flowchart, illustrating the progressive narrowing of document selections from a broad initial sweep of 3497 papers, trimmed down to 3048 after removing redundancies, eventually settling on 16 documents^[17–32]^ following elimination of irrelevant entries and stringent application of inclusion/exclusion rules.

### 3.2. Study characteristics

Table 1 lists the characteristics of the studies analyzed in the meta-study, which consisted of 16 studies involving 1335 cardiac surgery patients. Predominantly, investigations depicted cognitive training encompassing memory, attention, and recognition exercises, paired with supplementary health education to familiarize patients with surgical procedures and recommended pre- and post-operative precautions. Select studies went further to integrate psychological counseling, thereby offering emotional sustenance to patients. Delivery modes for cognitive training mostly centered around interactive seminars, workshops, conventional paper-and-pencil exercises, and digitally-mediated platforms. Demarcated disparities in the duration, frequency, delivery methods, and training schedules of cognitive training programs were prominent among the incorporated studies, as portrayed in Table 2.

**Table 1:** Characteristics of included studies

Author (year of publication)	Country	Sample size, n (Female gender, n)		Age, mean ± SD (yr)		Type of intervention		Type of disease	Data follow-up time (mo)	Outcomes
		EG	CG	EG	CG	EG	CG			
Paul, M. et al (1986)^[22]^	Canada	19 (7)	26 (9)	–	–	Relaxation training and cognitive therapy	Routine care	Coronary patients post-surgery	End of intervention	③④
Dao, TK. et al (2011)^[25]^	American	48 (11)	49 (10)	62.8 ± 11.8	64.2 ± 11.9	Cognitive behavioral therapy	Routine care	CABG surgery patients	End of intervention	③④⑤⑦
Hang, C. et al (2014)^[30]^	China	21 (−)	21 (−)	–	–	Cognitive function training	Routine rehabilitation care	Cardiac Intervention Patients	End of intervention	①②③
Doering, LV. et al (2016)^[17]^	American	33 (3)	20 (6)	63.90 ± 7.80	68.40 ± 9.00	Cognitive behavioral therapy	Routine care	Cardiac surgery patients	End of intervention	③
Beresnevaitė, M. et al (2016)^[20]^	Lithuania	43 (13)	46 (14)	56.70 ± 10.20	59.10 ± 11.10	Cognitive behavioral therapy	Routine care	Cardiac surgery patients	End of intervention	⑤
Jianfeng, Lv. et al (2016)^[23]^	China	38 (12)	37 (10)	52.40 ± 6.30	52.00 ± 6.20	Cognitive behavioral therapy	Routine care	percutaneous coronary intervention	End of intervention	③④⑤
Cao, MY. et al (2017)^[31]^	China	100 (31)	100 (32)	43.30 ± 7.30	44.10 ± 6.30	Cognitive behavioral therapy	Routine rehabilitation care	Patients undergoing coronary angiography plus percutaneous transluminal coronary angioplasty and endocardial electrophysiology plus radiofrequency ablation	End of intervention	⑦
Liu, JH. et al (2017)^[32]^	China	40 (18)	40 (17)	–	–	Cognitive behavioral therapy	Routine rehabilitation care	Patients undergoing elective coronary artery bypass graft surgery	End of intervention	②
Edwards, KS. et al (2020)^[21]^	American	80 (30)	66 (33)	82.52 ± 9.32	82.26 ± 7.45	Cognitive behavioral therapy	Routine care	Patients undergoing transcatheter aortic valve replacement	End of intervention	③④⑤
O’Gara, BP. et al (2020)^[24]^	American	20 (6)	20 (5)	70.00 ± 6.00	69.00 ± 7.00	Cognitive training program	Routine care	Cardiac surgery patients	End of intervention	①⑥⑦
Dong, NN. et al (2020)^[29]^	China	50 (−)	50 (−)	–	–	Cognitive function training	Routine rehabilitation care	Cardiac surgery patients	End of intervention	①②
Salimpour, M. et al (2022)^[18]^	Iran	26 (15)	24 (11)	51.81 ± 4.61	50.83 ± 7.70	Cognitive‐behavioral therapy	Routine care	Cardiac surgery patients	End of intervention	④
Butz, M. et al (2022)^[19]^	Germany	47 (8)	47 (13)	71.20 ± 4.70	73.00 ± 4.90	Cognitive training	Routine care	Patients scheduled for elective aortic or mitral valve replacement/reconstruction with or without CABG	Discharge	①③④⑥
Greaves, D. et al (2023)^[26]^	Australia	18 (4)	18 (2)	73.80 ± 6.40	72.60 ± 5.70	Computerized cognitive training	Routine care	Coronary artery bypass grafting	Discharge, 4-months and 6-months	②⑥
Butz, M. et al (2023)^[27]^	Germany	47 (8)	47 (13)	71.20 ± 4.70	73.00 ± 4.90	Cognitive training program	Routine rehabilitation care	Elderly patients for elective aortic or mitral valve replacements/reconstructions	The 3-month follow-up	⑤
Gerriets, T. et al (2023)^[28]^	Germany	47 (8)	47 (13)	71.20 ± 4.70	73.00 ± 4.90	Cognitive training program	Routine rehabilitation care	Elderly patients for elective aortic or mitral valve replacements/reconstructions	The 12-month follow-up	①⑤

① Postoperative cognitive dysfunction; ② Cognitive function; ③ Depression; ④ Anxiety; ⑤ Quality of life; ⑥ Postoperative delirium; ⑦ Length of postoperative hospitalization.

CABG = coronary artery bypass grafting; e.g., experimental group, CG = control group, NR = not reported.

**Table 2:** Characteristics of cognitive training programs

Author (year of publication)	Setting	Duration (wk)	Time (min)	Frequency (time/wk)	Methods of proceeding	Cognitive training content						
						Health promotion	Function training	Attention skills training	Psychological orientation training	Numeracy training	Expressive language skills training	Recognition training
Paul, M. et al(1986)^[22]^	Hospital	6	100	7	A	✔	✔	✔	✔			✔
Dao, TK. et al(2011)^[25]^	Hospital	NR	60	NR	A	✔	✔	✔	✔	✔		
Hang, C. et al (2014)^[30]^	Hospital	2	30–40	3	A	✔	✔	✔	✔			✔
Doering, LV. et al (2016)^[17]^	Hospital	8	60	1	C	✔	✔	✔				✔
Beresnevaitė, M. et al (2016)^[20]^	Household	36	NR	NR	A	✔	✔	✔	✔			✔
Jianfeng, Lv. et al (2016)^[23]^	Hospital	NR	NR	NR	A	✔	✔		✔			✔
Cao, MY. et al (2017)^[31]^	Hospital	NR	NR	NR	A	✔			✔			
Liu, JH. et al (2017)^[32]^	Hospital	2	30	NR	A	✔	✔		✔		✔	✔
Edwards, KS. et al (2020)^[21]^	Hospital	NR	NR	NR	A	✔	✔	✔		✔		✔
O’Gara, BP. et al (2020)^[24]^	Hospital	4	15	14	B		✔	✔	✔			✔
Dong, NN. et al (2020)^[29]^	Hospital	1	60	7	A		✔	✔		✔	✔	✔
Salimpour, M. et al (2022)^[18]^	Hospital	4	NR	2	A	✔	✔	✔	✔			✔
Butz, M. et al (2022)^[19]^	Hospital	3	36	6	C	✔	✔	✔	✔		✔	✔
Greaves, D. et al (2023)^[26]^	Household	12	45–60	3	B		✔	✔				✔
Butz, M. et al (2023)^[27]^	Hospital	2	NR	7	C		✔	✔	✔			✔
Gerriets, T. et al (2023)^[28]^	Hospital	3	36	6	C		✔	✔	✔			✔

A = conference format or classroom model, B = via computer or communication device, C = paper and pencil-based training, NR = not reported.

### 3.3. Risk of bias in included studies

**Figure 2.**
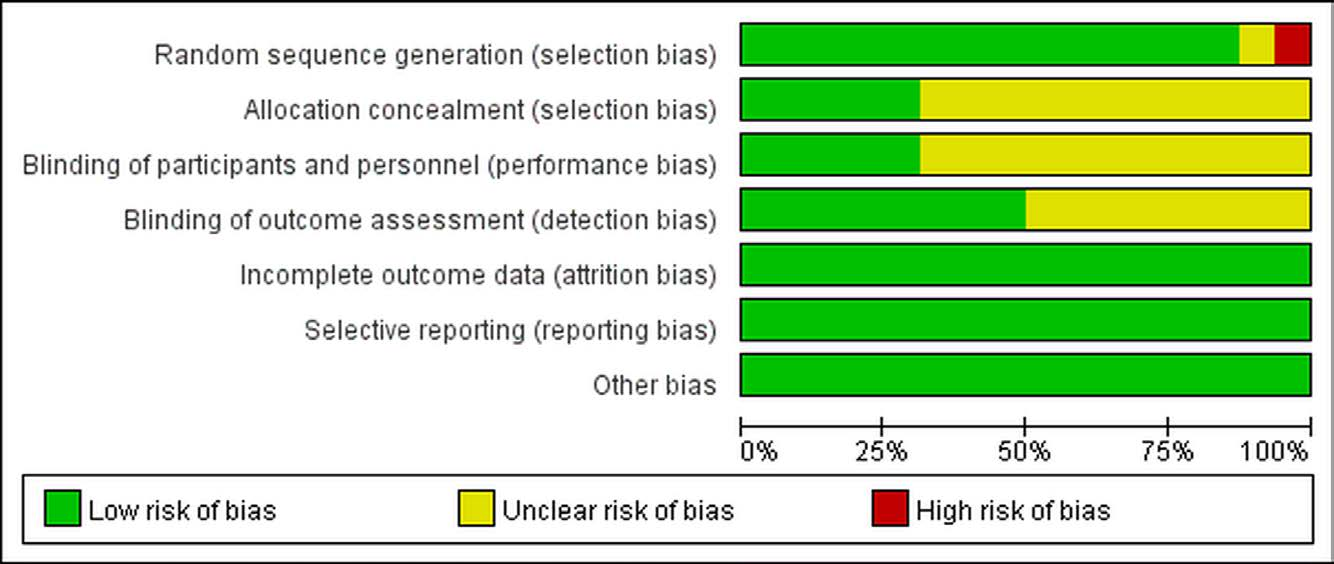
Risk of bias graph. The overall literature quality of the included studies was good.

**Figure 3.**
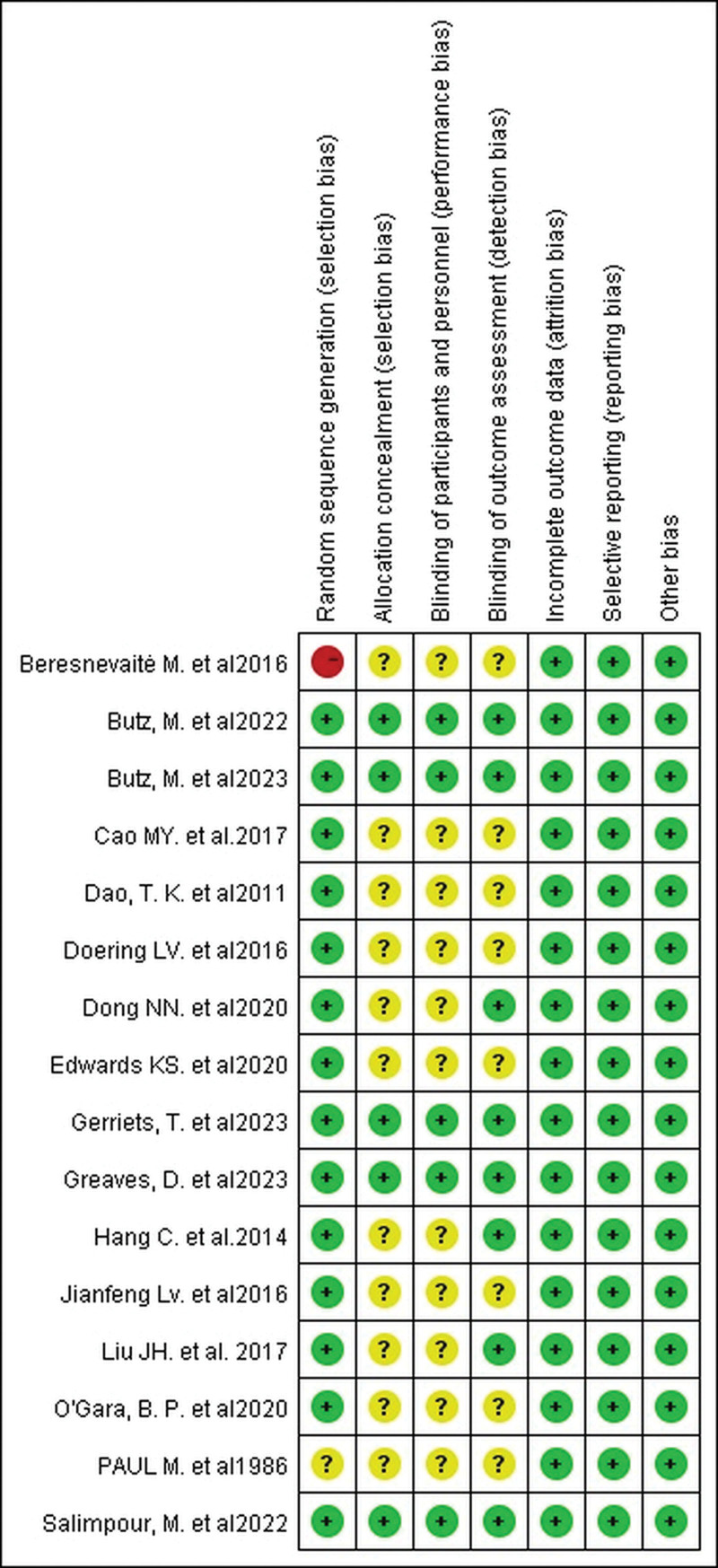
Risk of bias summary. Five studies had a low risk of risk of bias for all entries in the risk of bias assessment.

The included studies were at low risk of attrition bias, reporting bias, and other biases. Although most of them embodied randomized controlled trials, most of them did not specifically describe their protocol allocation methods and blinding details, as shown in Figure 2. There were 5 high-quality literatures in this study, and all 7 bias analysis entries were at low risk, as shown in Figure 3. Overall, the literature included in this study was of good quality, the overall risk of bias was low, and the reliability of the Meta-analysis results was high.

### 3.4. Primary outcomes

#### 3.4.1. POCD

**Figure 4.**
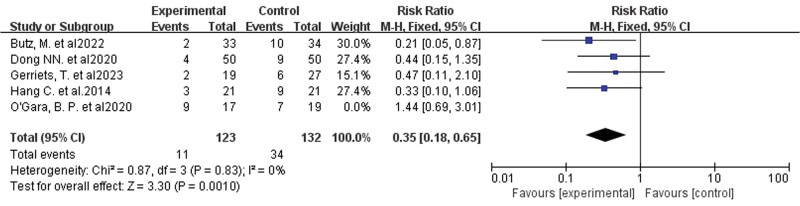
Forest plot of the effect of cognitive training on the incidence of POCD. The combined effect sizes showed a significantly lower incidence of POCD in the intervention group compared to the control group. POCD = postoperative cognitive dysfunction.

**Figure 5.**
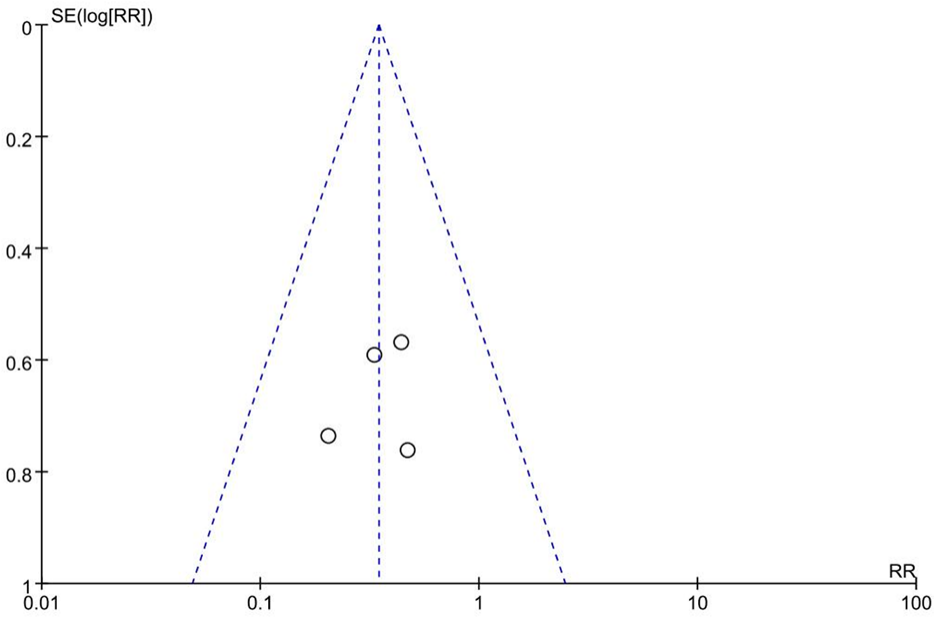
Funnel plot of the effect of cognitive training on POCD incidence. No significant publication bias was shown. POCD = postoperative cognitive dysfunction.

Upon aggregation and critical evaluation of 5 distinct studies^[19,24,28–30]^ measuring POCD prevalence, significant heterogeneity (*I*^2^ = 57%) surfaced. Through one-by-one elimination, the root of such discrepancy was localized to the O’Gara et al. study.^[24]^ The sources of heterogeneity were excluded and the remaining 4 studies (involving 255 patients)^[19,28–30]^ were analyzed together, showing complete disappearance of heterogeneity (*I*^2^ = 0%). Applying a fixed-effects model to analyze cognitive training’s impact on POCD incidence confirmed its substantial decrease (RR 0.35, 95% CI 0.18–0.65, *P* = .001, Fig. 4). The funnel plot (Fig. 5) did not reveal significant publication bias.

#### 3.4.2. Cognitive function

**Figure 6.**
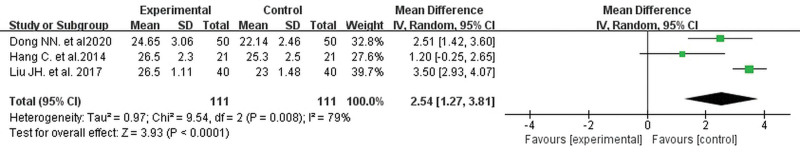
Forest plots of the effects of cognitive training on cognitive function. Combined effect sizes showed significant improvement in cognitive functioning in the intervention group compared to the control group.

Four studies^[26,29,30,32]^ reported on cognitive function. The Greaves et al study,^[26]^ regrettably bereft of the specific numeric representation, suggested the negligible influence of computerized cognitive training on holistic cognitive performance across discharge, 4-month, and 6-month checkpoints. Given the inconsistency of the cognitive function evaluation tools used in each of the remaining three studies (involving 222 patients), a random-effects model was inherently used, and the results showed that cognitive training significantly improved cognitive function in patients after cardiac surgery (MD 2.54, 95% CI 1.27–3.81, *P* < .001, Fig. 6). Sensitivity analysis and publication bias tests were not performed owing to the small amount of research literature.

#### 3.4.3. Postoperative delirium

**Figure 7.**
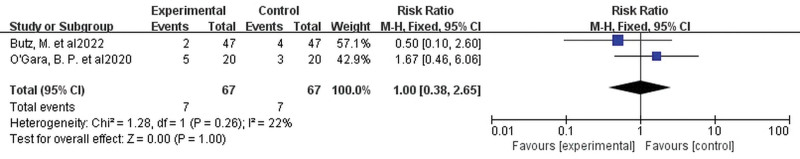
Forest plot of the effect of cognitive training on postoperative delirium. The combined effect sizes showed no statistically significant difference in postoperative delirium between the intervention and control groups.

Three distinct studies^[19,24,26]^ touched upon the relationship between postoperative delirium and cognitive training. The Greaves et al research,^[26]^ despite lacking concrete numerical expression, reported a trivial association between pre-surgical computational cognitive training and delirium occurrence following Coronary Artery Bypass Grafting operations (odds ratio = 1.25, 95% CI [0.30, 5.24], *P* = .76). The other 2 (involving 134 patients) combined analyses sporting reduced heterogeneity (*I*^2^ = 22%) and capitalizing on a fixed-effects model concluded an insignificant impact of cognitive training on post-cardiac surgical delirium (RR 1, 95% CI 0.38–2.65, *P* = 1.00, Fig. 7). Sensitivity analysis and publication bias tests were not performed owing to the small amount of research literature.

### 3.5. Secondary outcomes

#### 3.5.1. Quality of life

Consolidating 6 studies^[20,21,23,25,27,28]^ discussing postoperative quality of life, stark inconsistencies punctuated assessment tools and scoring methodologies. Gerriets et al’s study^[28]^ harnessed the SF-36 scale sans concrete scores, yet disclosed significant enhancement in both physical (*P* = .005) and mental (*P* = .018) realms following cognitive training. Another study helmed by Jianfeng et al^[23]^ applying the Coronary Artery Revascularization Outcome Questionnaire, uncovered significant betterment in patients” quality of life thanks to cognitive training. However, invoking the SF-12 scale, Dao et al^[25]^ determined that cognitive training exerted no remarkable influence on quality of life.

**Figure 8.**
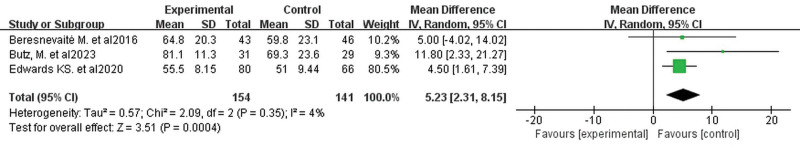
Forest plot of the effect of cognitive training on quality of life-mental component. The combined effect sizes showed statistically significant differences in quality of life-mental components between the intervention and control groups.

**Figure 9.**
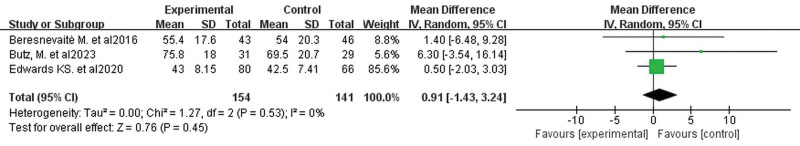
Forest plot of the effect of cognitive training on quality of life-physical component. The combined effect sizes showed no statistically significant difference in the quality of life-physical component between the intervention and control groups.

Utilizing both SF-12 and SF-36 inventories, physical and mental constituents were equally assessed and computed in these three studies^[20,21,27]^ (involving 295 patients). Henceforth, a random effects model was used for the combined analysis, and the results showed that cognitive training significantly improved the mental component (MD 5.22, 95% CI 2.32–8.13, *P* < .001, Fig. 8), but had no significant effect on the physical component (MD 0.91, 95% CI −1.43 to 3.24, *P* = .45, Fig. 9). Sensitivity analysis and publication bias tests were not performed owing to the small amount of research literature.

#### 3.5.2. Depression

**Figure 10.**
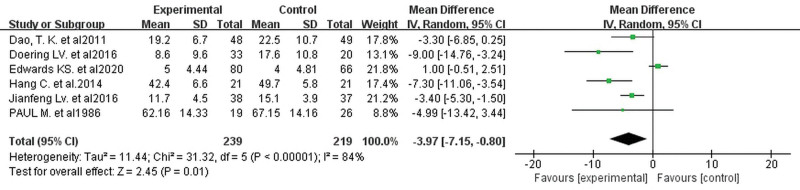
Forest plots of the effects of cognitive training on depression. Combined effect sizes showed significantly lower depression scores in the intervention group compared to the control group.

**Figure 11.**
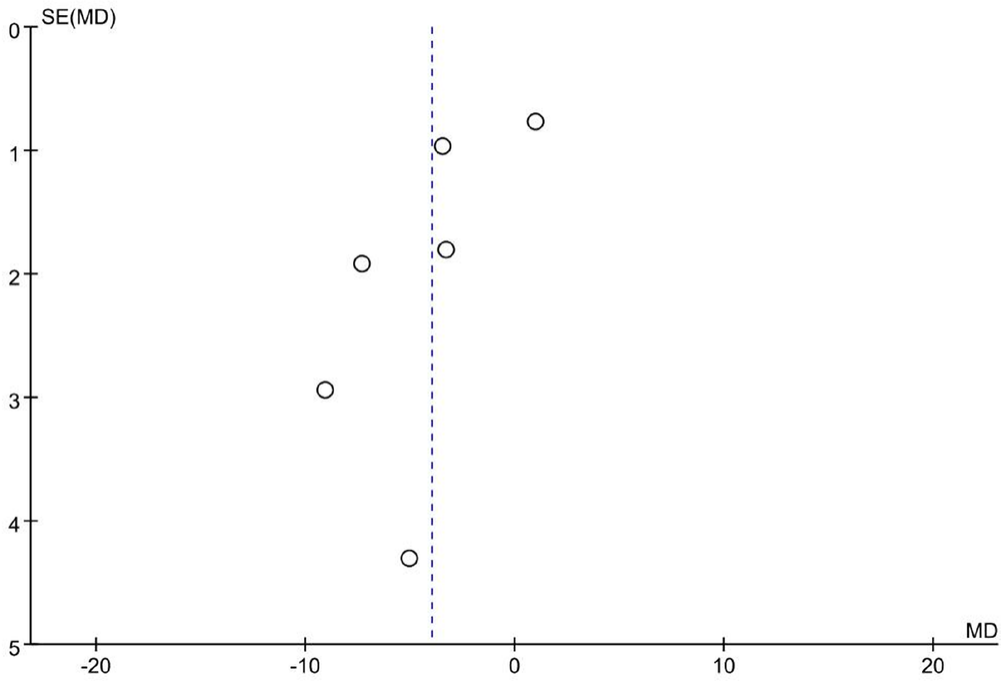
Funnel plot of the effect of cognitive training on depression. No significant publication bias was shown.

Contributing 6 distinct studies^[17,21–23,25,30]^ (involving 458 patients) furnished evaluative data regarding post-cardiac surgical depressive symptomatology, assessment instruments varied inconsistently, however, lower scores never corresponded to improved patient circumstances. Succumbing to this discrepancy, a random-effects model synopsized the data, unmasking that cognitive training noticeably alleviated depressive symptoms in comparison to controls (MD −3.97, 95% CI −7.15 to −0.80, *P* = .01, Fig. 10). The funnel plot (Fig. 11) did not reveal significant publication bias.

#### 3.5.3. Anxiety

**Figure 12.**
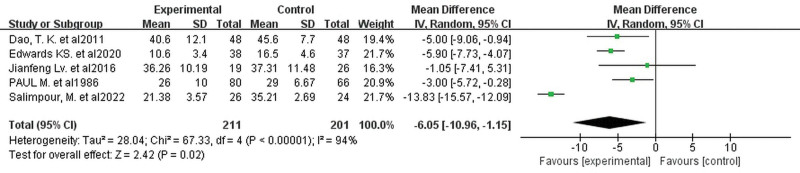
Forest plot of the effect of cognitive training on anxiety. Combined effect sizes showed significantly lower anxiety scores in the intervention group compared to the control group.

**Figure 13.**
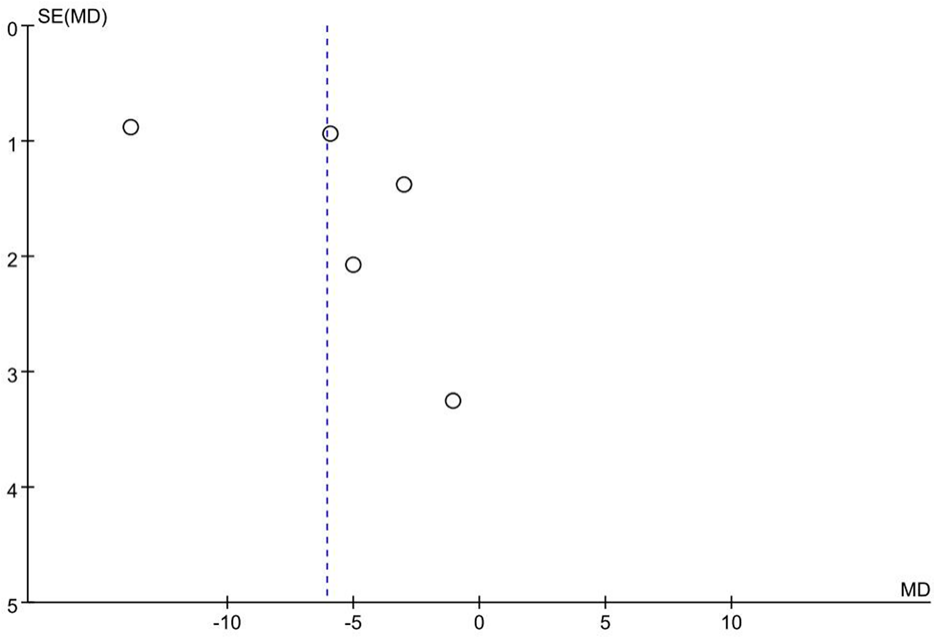
Funnel plot of the effect of cognitive training on anxiety. Shows significant publication bias.

It was not possible to obtain data specific to Butz et al^[19]^ The results of this study indicated that there was no significant difference between the cognitive training and non-training counterpart studies in reducing anxiety in cardiac surgery patients (*P* = .063). Data on anxiety scores in the 5 studies^[18,21–23,25]^ (involving 412 patients) were extracted, due to differences in the use of evaluation scales in the studies, but all of them were in a better state with lower scores. Therefore, a random effects model was used and the results showed that cognitive training significantly improved anxiety status in patients after cardiac surgery (MD −6.05, 95% CI −10.96 to −1.15, *P* = .02, Fig. 12). The funnel plot (Fig. 13) shows clear publication bias.

#### 3.5.4. Length of postoperative hospitalization

**Figure 14.**
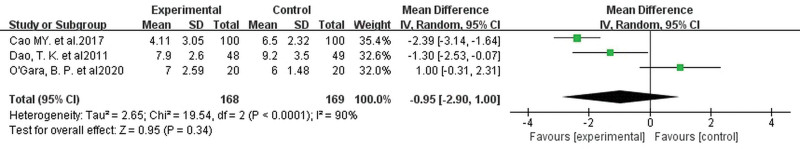
Forest plot of the effect of cognitive training on postoperative length of stay. The combined effect sizes showed no statistically significant difference in the length of postoperative hospitalization between the intervention and control groups.

Harnessing a random-effects model to tackle stark heterogeneity (*I*^2^ = 90%) among three studies^[24,25,31]^ (involving 337 patients) addressing postoperative hospitalization time frame, no identifiable origins were discovered utilizing the one-by-one elimination tactic. Results implied that cognitive training failed to generate significant modifications to patients’ postoperative hospitalization durations after cardiac surgery (MD −0.95, 95% CI −2.90 to 1.00, *P* = .34, Fig. 14). Sensitivity analysis and publication bias tests were not performed owing to the small amount of research literature.

### 3.6. GRADE assessment of study outcomes

The GRADE assessment of the outcome measures indicates that the final certainty of evidence for postoperative cognitive dysfunction and depression is moderate. In contrast, the evidence for cognitive function, postoperative delirium, quality of life (physical and mental components), and anxiety is of lower certainty. The certainty of evidence regarding the length of postoperative hospitalization is rated as very low (Table 3). The main factors contributing to the downgrading of evidence include small sample sizes, inconsistencies in measurement tools, and risk of bias.

**Table 3:** Summary of findings table: GRADE assessment of outcome measures

Outcome	Study design	No. of studies (participants)	Certainty of evidence (initial rating)	Key findings	Reasons for downgrading	Certainty of evidence (final rating)
Postoperative cognitive dysfunction	RCT	4 studies (255 patients)	High	RR 0.35 (95% CI 0.18–0.65)	Imprecision (small sample size)	Moderate
Cognitive function	RCT	3 studies (222 patients)	High	MD 2.54, (95% CI 1.27–3.81)	Inconsistency (inconsistent measurement tools for outcome indicators), imprecision (small sample size)	Low
Postoperative delirium	RCT	2 studies (134 patients)	High	RR 1 (95% CI 0.38–2.65)	Slight inconsistency (*I*^2^ = 22%), imprecision (small sample size)	Low
Quality of life (physical component)	RCT	3 studies (295 patients)	High	MD 0.91 (95% CI −1.43 to 3.24)	Risk of bias, inconsistency (inconsistent measurement tools for outcome indicators), imprecision (small sample size)	Very low
Quality of life (mental component)	RCT	2 studies (295 patients)	High	MD 5.22 (95% CI 2.32–8.13)	Risk of bias, inconsistency (inconsistent measurement tools for outcome indicators), and imprecision (small sample size)	Very low
Depression	RCT	6 studies (485 patients)	High	MD −3.97 (95% CI −7.15 to −0.80)	Risk of bias, Inconsistency (inconsistent measurement tools for outcome indicators)	Moderate
Anxiety	RCT	5 studies (412 patients)	High	MD −6.05 (95% CI –10.96 to –1.15)	Inconsistency (inconsistent measurement tools for outcome indicators), publication bias	Low
Length of postoperative hospitalization	RCT	3 studies (337 patients)	High	MD −0.95 (95% CI −2.90 to 1.00)	Risk of bias, inconsistency (*I*^2^ = 90%), imprecision (small sample size)	Very low

RCT = randomized controlled trial.

## 4. Discussion

Cognitive training has been identified as a potential therapeutic approach to promote the physical and mental rehabilitation of patients following cardiac surgery. According to this study, cognitive training has shown promise in decreasing the prevalence of POCD, enhancing cognitive abilities, and reducing depressive and anxious symptoms in individuals who have undergone cardiac surgery. These discoveries hold significance in improving the postoperative rehabilitation experience and minimizing postoperative complications. Notably, existing data do not substantiate the claim that cognitive training influences postoperative delirium, duration of hospital stay, or overall quality of life for patients recovering from cardiac surgery.

This research reveals that cognitive training effectively enhances cognitive function in patients following cardiac surgery. A meta-analysis incorporating 17 studies and 679 participants diagnosed with Parkinson’s disease revealed a notable impact of cognitive training on global cognitive capacity, particularly among those exhibiting mild cognitive decline.^[33]^ Similarly, Tarasova et al’s investigation illustrated the favorable consequences of cognitive training interventions on post-cardiac surgical patients, while highlighting superior outcomes associated with multi-tasking cognitive exercises in ameliorating cognitive performance.^[34]^ Analogous advantages of cognitive enhancement have been documented in patients afflicted by post-stroke cognitive deficiencies and in cognitively intact older adults^[35,36]^ By capitalizing on neural plasticity, cognitive training fortifies cerebral networks via persistent drills and iterations, thereby bolstering the brain’s aptitude to assimilate, organize, and evaluate exogenous information, cultivating higher-order critical thinking skills, and concurrently mitigating stress and negative emotional responses during the learning process.^[37]^ Therefore, cognitive training represents a comprehensive strategy for potentiating neuroplasticity and reinforcing cognitive faculties.

POCD and delirium have evolved into crucial predictors during the postoperative phase, as evidenced in this study. Specifically, cognitive training has proven efficacy in lowering the incidence of POCD, although its influence on postoperative delirium remains ambiguous. Concurring with these observations, a meta-analysis conducted by Li et al, encompassing 11 randomized controlled trials and 1045 postoperative subjects, established that preemptive cognitive training significantly curtailed POCD frequency, yet had negligible bearing on postoperative delirium prevalence.^[38]^ Acute deterioration in cognitive processing velocity commonly manifests in postoperative patients, notably amongst those having undergone cardiovascular procedures, consequently impeding their restoration of functional capabilities. Nevertheless, recurrent cognitive training can counteract this downtrend in processing rate,^[37]^ thereby accelerating recuperation, diminishing POCD incidence, curtailing accident risks in patients, elevating patients’ quality of life, and simultaneously easing burdens on health care systems.

Cognitive training constitutes a pivotal component in the modulation of affective states. Empirical evidence supports the beneficial impacts and safety profile of cognitive training when employed in managing adult depression and anxiety disorders.^[39]^ Nonetheless, discrepancies arise regarding the utility of cognitive training in attenuating anxiety and depressive symptomatology within cohorts grappling with malignant neoplasm. Yan et al’s investigation incorporated nine randomized control trials, totaling 666 female breast cancer patients, reporting insignificant improvements in anxiety and depression after cognitive therapy implementation.^[40]^ Conjectured explanatory factors include distinctive attributes inherent to cancer populations and potentially compromised sensitivity of applied measurement apparatuses eliciting biased evaluations. Cognitive training enables transformative perception modifications concerning encountered experiences via reconstructive processes. Complementary strategies such as emotion regulation training foster comprehension and management of emotional reactions while relaxation techniques facilitate effective reduction of physiological and psychological strains, ultimately alleviating anxiety and depressive incidents.^[41]^

Presently, this study fails to yield conclusive evidence detailing the ramifications of cognitive training on quality of life for patients subjected to cardiac surgeries. Disparities in adopted assessment methodologies across eligible investigations coupled with inconsistent scoring algorithms rendered synthesis untenable. Meta-analytic outputs disclosed substantial distinctions solely within the mental dimension of quality of life, whilst physical components remained unaffected between experimental and control groups. Such findings possibly reflect the predominantly neurologically oriented focus characterizing many studies, which frequently overlooked corollary physical exercise aspects. Moreover, Gates et al’s work implies the limited impact of digitized cognitive regimens upon existential qualities among geriatric patients experiencing minimal cognitive debilitations.^[42]^ In the future, high-quality, multi-sample, and multi-center studies are needed to further determine the impact of cognitive training on patients” quality of life.

This meta-analysis showed that the difference in postoperative length of stay between the cognitive function training group and the control group was not statistically significant (*P* = .34). The quality of evidence for this outcome indicator is low because only 3 studies reported postoperative hospitalization time.

Diverse modalities encompass contemporary cognitive training, inclusive of paper-and-pen-based exercises, digital-platform mediated programs, multiplex cognitive tasks, sensorimotor integrative training, virtual reality applications, and psychotherapeutic methods paired with biosensor feedback. Currently, computer-based cognitive training is the dominant modality, which can be trained at home and is convenient, practical, and versatile for patients. Future large-sample, high-quality, multicenter randomized controlled trials are needed to determine which type of cognitive training has the best effect on patients.

### 4.1. Limitations

Some of the limitations of this study are: first, a few outcome indicators of some trials could not be extracted directly from the data, but rather the data were processed through statistical methods, which may lead to biased results. Second, only English and Chinese literature were included in this study, which may be biased. Third, some of the results summarized in this study had a high degree of heterogeneity, which may be due to differences in cognitive training styles, training frequency, and duration. Fourth, this study did not explore the effectiveness of cognitive training based on differences in training modalities, training locations, and surgical approaches. Fifth, some of the findings in this study may have some bias because the number of included studies was too small to be analyzed for publication bias. Future high-quality, large-sample, multicenter randomized trial studies are needed to explore the effects of cognitive training on cardiac surgery patients.

## 5. Conclusions

This study is based on low- to moderate-quality evidence that cognitive training considerably enhances cognitive functionality, lessens the occurrences of POCD, and attenuates symptoms of both anxiety and depression among cardiac surgical patients. Based on limited research, however, prevents decisive conclusions regarding how cognitive training affects patients’ general well-being, postoperative delirious conditions, and hospitalization periods.

## Author contributions

**Data curation:** Rongxiang Zhang, Chenyang Zhu.

**Formal analysis:** Shiqi Chen.

**Methodology:** Rongxiang Zhang, Chenyang Zhu, Shiqi Chen, Feng Tian, Pingping Huang, Yuan Chen.

**Project administration:** Feng Tian.

**Software:** Rongxiang Zhang, Shiqi Chen, Feng Tian, Yuan Chen.

**Supervision:** Rongxiang Zhang, Chenyang Zhu, Shiqi Chen, Yuan Chen.

**Writing – original draft:** Rongxiang Zhang.

**Writing – review & editing:** Shiqi Chen, Feng Tian, Pingping Huang, Yuan Chen.

## Supplementary Material


