# Supplementary material for: Effects of cognitive training on cognitive function in patients after cardiac surgery: A systematic review and meta-analysis of randomized controlled trials
Source: Medicine (Baltimore). 2024 Nov 1;103(44):e40324. doi: 10.1097/MD.0000000000040324 (PMC11537618; doi:10.1097/MD.0000000000040324)
Supplement: Supplementary file 1 [file medi-103-e40324-s001.docx]

Supplementary Material

Pubmed Search strategy:

1. Cardiac Surgical Procedures[MeSH Terms]
2. Cardiovascular Surgical Procedures[MeSH Terms]
3. Cardiac Surgical Procedures[Title/Abstract]
4. Cardiovascular Surgical Procedures[Title/Abstract]
5. Cardiac surgery[Title/Abstract]
6. heart surgery[Title/Abstract]
7. valve surgery[Title/Abstract]
8. Coronary Artery Bypass[Title/Abstract]
9. coronary bypass grafting[Title/Abstract]
10. OR/ 1 - 9
11. Cognitive training[MeSH Terms]
12. cognitive behavior therapies[MeSH Terms]
13. Cognitive training[Title/Abstract]
14. cognitive behavior therapies[Title/Abstract]
15. Cognitive Rehabilitation[Title/Abstract]
16. (Brain Training[Title/Abstract]
17. Memory Training[Title/Abstract]
18. Cognitive therapy[Title/Abstract]
19. Heart Valve Diseases[Title/Abstract]
20. OR/ 11 - 19
21. 10 AND 20
